# Reversible Immobilization of Lipases on Heterofunctional Octyl-Amino Agarose Beads Prevents Enzyme Desorption

**DOI:** 10.3390/molecules21050646

**Published:** 2016-05-16

**Authors:** Nazzoly Rueda, Tiago L. Albuquerque, Rocio Bartolome-Cabrero, Laura Fernandez-Lopez, Rodrigo Torres, Claudia Ortiz, Jose C. S. dos Santos, Oveimar Barbosa, Roberto Fernandez-Lafuente

**Affiliations:** 1 Departamento de Biocatalisis, Instituto de Catálisis-CSIC; C/ Marie Curie 2, Campus UAM-CSIC, Madrid 28049, Spain; nazzoly@gmail.com (N.R.); tiagotla1@gmail.com (T.L.A.); rociobartolomecabrero2010@gmail.com (R.B.-C.); laura_valde95@hotmail.com (L.F.-L.); jscleiton@gmail.com (J.C.S.d.S.); 2 Escuela de Química, Grupo de investigación en Bioquímica y Microbiología (GIBIM), Edificio Camilo Torres 210, Universidad Industrial de Santander, Bucaramanga 680002, Colombia; rodrigo.torres@ecopetrol.com.co; 3 Departamento de Engenharia Química, Universidade Federal Do Ceará, Campus Do Pici, CEP 60455-760 Fortaleza, Brazil; 4 Escuela de Microbiología, Universidad Industrial de Santander, Bucaramanga 680002, Colombia; ortizc@uis.edu.co; 5 Departamento de Química, Facultad de Ciencias, Universidad del Tolima, Ibagué 546, Colombia; oveimar@gmail.com

**Keywords:** heterofunctional supports, octyl supports, interfacial activation of lipases, ion exchange, enzyme hyperactivation, reversible immobilization

## Abstract

Two different heterofunctional octyl-amino supports have been prepared using ethylenediamine and hexylendiamine (OCEDA and OCHDA) and utilized to immobilize five lipases (lipases A (CALA) and B (CALB) from *Candida antarctica*, lipases from *Thermomyces lanuginosus* (TLL), from *Rhizomucor miehei* (RML) and from *Candida rugosa* (CRL) and the phospholipase Lecitase Ultra (LU). Using pH 5 and 50 mM sodium acetate, the immobilizations proceeded via interfacial activation on the octyl layer, after some ionic bridges were established. These supports did not release enzyme when incubated at Triton X-100 concentrations that released all enzyme molecules from the octyl support. The octyl support produced significant enzyme hyperactivation, except for CALB. However, the activities of the immobilized enzymes were usually slightly higher using the new supports than the octyl ones. Thermal and solvent stabilities of LU and TLL were significantly improved compared to the OC counterparts, while in the other enzymes the stability decreased in most cases (depending on the pH value). As a general rule, OCEDA had lower negative effects on the stability of the immobilized enzymes than OCHDA and while in solvent inactivation the enzyme molecules remained attached to the support using the new supports and were released using monofunctional octyl supports, in thermal inactivations this only occurred in certain cases.

## 1. Introduction

Immobilization is a requirement for most uses of lipases as industrial biocatalysts in the chemical industry [1,2,3,4,5,6,7,8,9]. This allows the subsequent separation of the enzymes from the reaction medium upon completion of the reactions, and, if the biocatalysts are stable enough, their reuse [1,2,3,4,5,6,7,8,9]. A proper immobilization also may improve other enzyme properties, like activity, selectivity or resistance to inhibitors [1,2,3,4,5,6,7,8,9]. Therefore, an efficient lipase immobilization system may be critical in the design of lipase-biocatalysts. Moreover, the interest of the immobilization may increase if it is coupled to the purification of the enzyme [10]. In this regard, immobilization of lipases on octyl-agarose (OC) is a simple method to achieve the rapid one step immobilization, stabilization, purification and hyperactivation of many lipases [11]. The lipase immobilization on these supports is based on the peculiar mechanism of action of lipases: interfacial activation [12,13]. Most lipases have a closed conformation, where the active center is secluded from the reaction medium by a polypeptide chain called lid or flat, which has a hydrophobic internal face that interacts with the hydrophobic surroundings of the active center. The lid may be very small, not really secluding the active center of the lipase from the medium as it is the case of the lipase B from *Candida antarctica* [14], or be something more complicated, like in the case of the lipase from *Bacillus thermocanetulatus*, that has a double lid that simultaneously moves during the interfacial activation [15]. In the open conformation, the lid moves, exposing a large hydrophobic pocket to the medium so that the substrate can accede to the active site. This open form is unstable in aqueous and homogenous medium, but becomes stabilized by adsorption on hydrophobic surfaces, like drops of oils [16,17,18]. This lipase adsorption occurs on any hydrophobic surface, like a hydrophobic protein [19], another lipase in its open form [20,21,22], or a hydrophobic support [23,24,25]. This way, the lipases are selectively immobilized on hydrophobic supports at low ionic strength via their interfacial activation *versus* the support surface, yielding the stabilized open form of the lipase as it has been recently shown [13]. There are some papers that state that this open and adsorbed on hydrophobic surfaces lipase form is more stable than the not adsorbed lipase [26,27], and in fact lipases immobilized on hydrophobic supports tend to be more stable than immobilized lipases stabilized by multipoint covalent attachment [28,29,30].

In spite of its advantages, the immobilization of lipases on hydrophobic supports via interfacial activation has an important drawback: the enzyme becomes released from the support in the presence of detergents or high concentrations of hydrophobic organic cosolvents [31,32], reducing the range of applications of these biocatalysts. Although the enzymes are adsorbed via the pocket formed by the surroundings of the active center, this is not blocked and crosslinked lipases immobilized on octyl-agarose have been successfully utilized in the hydrolysis of fish oil in the presence of organic solvents [31,32].

The use of heterofunctional octyl-glyoxyl beads has been proposed as a way to avoid this enzyme release during operation [33,34,35,36,37,38,39,40]. However, some enzyme molecules that were not covalently attached via the glyoxyl groups and enzymes from some sources resulted partially inactivated by the alkaline incubation required [36]. Moreover, the immobilization protocol using octyl-glyoxyl support is an irreversible method, negating the possibility of reusing the support after enzyme inactivation. Reversibility, if the enzyme is not desorbed during operation, is an advantage for an immobilization method, particularly for an enzyme that may be used in sectors as diverse as food technology (manipulation of oils and fats), energy (biodiesel) or fine chemistry, in some cases the price of the biocatalyst may be critical for the economical suitability of the process [41] and the support reuse may be a further advantage.

In this paper, a new strategy for the use of octyl-agarose to immobilize, purify, stabilize and hyperactivate lipases, and also useful in the presence of organic solvents or detergents while keeping the reversibility of the immobilization, is proposed using the concept of heterofunctional supports [42]. It is based on the conversion of octyl-agarose in a heterofunctional octyl-amino agarose support. In this case, we wish to initialize the enzyme immobilization by the interfacial activation of lipases versus the octyl layer (keeping the hyperactivation and selectivity of the lipase adsorption) [13] and after, by changing the experimental conditions and taking advantage of the proximity of the support and enzyme surfaces, get some ionic bridges between the cation residues in the support and the anion groups on the enzyme surface (avoiding the desorption of the enzyme if the hydrophobic interactions are broken by organic solvents or non-ionic detergents). Crosslinked octyl agarose beads have some diols (derived from the opening of the epoxy moieties during the support preparation) that may be easily oxidized to glyoxyl groups with periodate [34], and may be later modified with a diamine to introduce a primary and a secondary amino group. This support will be useful as an anion exchanger [43].

Considering that most proteins may be ionically exchanged on anion exchangers at pH 7 [44,45], we should look for pH or ionic strength values where the lipase cannot become ionically exchanged in the support, to ensure that the enzyme will firstly immobilize via interfacial activation. However, after the first enzyme immobilization, due to the change of the conditions and to the proximity of the enzyme and the support surfaces, the immobilized enzyme molecules and the support may establish some ionic bridges. This ionic exchange will only involve the area of the enzyme surrounding the active center. If this mixed adsorption is achieved, the enzyme molecules will not be desorbed from the support in organic medium, or in the presence of non-ionic detergents, while the reversibility will be maintained (e.g., using ionic detergents or guanidine the enzymes may be released). A multi-ionic exchange is required to prevent the enzyme desorption once the hydrophobic interactions are annulled because the enzyme will not remain immobilized on a support via a single ionic bridge [46,47].

The strategy has been assayed using two different octyl-amino supports, ethylendiamine (EDA) and hexylendiamine (HDA), to modify the octyl-agarose support. Using EDA, both amino groups will be under the octyl layer, one of the groups will have a pK under 7 (the primary one) while the secondary amino group has a pK over 10 and will be closer to the support surface [43]. Using HDA, the primary amino group will be almost at the same level than the octyl layer, while the pK of both groups will be over 10. Six different enzymes have been assayed: lipases A (CALA) and B (CALB) [14,48,49,50] from *Candida antarctica*, lipases from *Thermomyces lanuginosus* (TLL) [51], from *Rhizomucor miehei* (RML) [52,53] and from *Candida rugosa* (CRL) [27,50]. Moreover, the phospholipase Lecitase Ultra [54,55] has also been included in these studies. It is a commercial chimeric enzyme constructed from the gene of the lipase from *Thermomyces lanuginosus* and that of the phospholipase from *Fusarium oxysporum* [56]*.*

## 2. Results

### 2.1. Immobilization on EDA and HDA Supports

**Figure 1:**
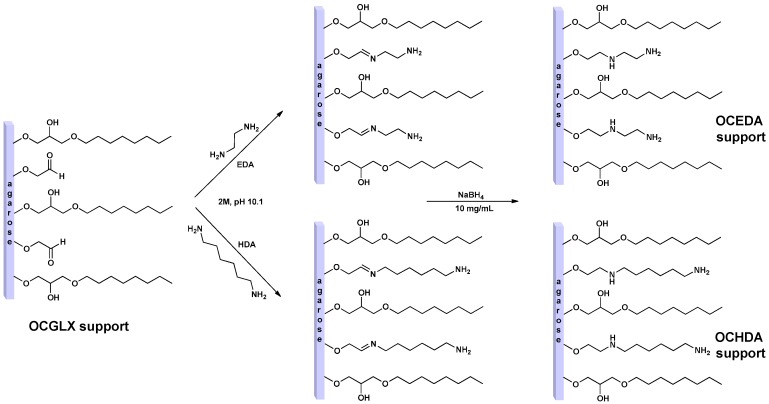
Preparation of different amino supports. Experiments have been performed as described in Section 2.

The main objective of this paper was to develop a method that enables the immobilization of lipases via interfacial activation, and only later establishing some ionic bonds between the immobilized enzyme and the support using octyl-amino supports (Figure 1). Thus, first we analyzed the adsorption of the lipases on pure amino supports. None of the lipases became immobilized on the amino supports at pH 5 in 50 mM sodium acetate (results not shown), therefore these conditions seem adequate for the first immobilization of the enzyme on octyl-amino supports.

**Figure 2:**
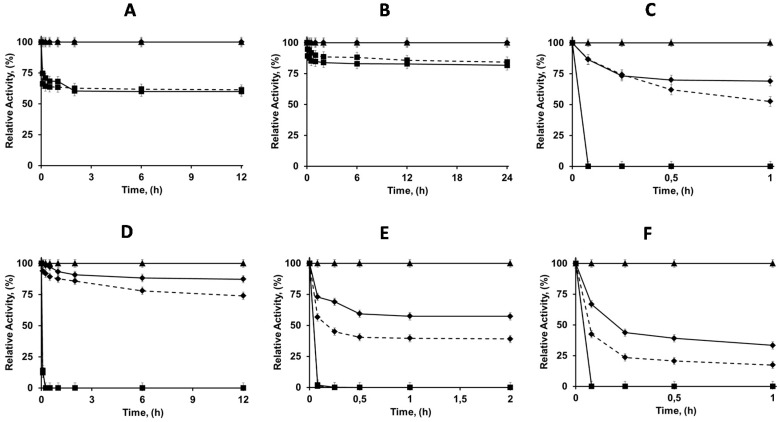
Immobilization courses of different lipases on amino agarose supports, EDA (solid black line) and HDA (dashed line). Immobilizations were performed in 5 mM buffer sodium phosphate at pH 7 and 25 °C. Other specifications are described in Section 2. (**A**) CALA; (**B**) CALB; (**C**) CRL; (**D**) RML; (**E**) TLL and (**F**) LU. Rhombus (suspension), square (Supernatant), triangle (Soluble enzyme).

On the other hand, it seemed interesting to immobilize the enzymes in pure aminated supports, just as a second reference to compare the properties of the immobilized enzymes (the first one is the OC support). Figure 2 shows the results. Even at pH 7 and low ionic strength, immobilization of the lipases was quite slow on EDA and HDA supports. Thus, only 40% of the CALA activity was immobilized on both aminated supports, maintaining 100% of the activity. Using CALB; the immobilization yield was even lower, with just 20%, once more maintaining 100% of the activity. The immobilization of CRL was complete in both supports, but using HDA (which primary amino group has a higher pK) the activity decreased to around 50% while this decrease was only of around 30% using EDA. This result was repeated using RML, with a decrease of activity to 83% or 75% depending on the support (EDA or HDA respectively). TLL was also fully immobilized on both supports; the activity decreased to 55% using EDA and to 40% using HDA. LU was also fully immobilized, but the cost of activity was the highest one, only 33% of enzyme activity was expressed in the EDA support and 17% in the HDA support.

Thus, five of the enzymes could be immobilized (at least partially) on the amino supports (for CALB immobilization was too low). The activity was, in the best cases, maintained, and generally suffered a decrement, usually higher using HAD, that has a longer spacer arm and a higher ion exchanger capacity. These preparations were used as references in further studies.

### 2.2. Immobilization of Enzymes in OC, OCEDA and OCHDA Support

**Figure 3:**
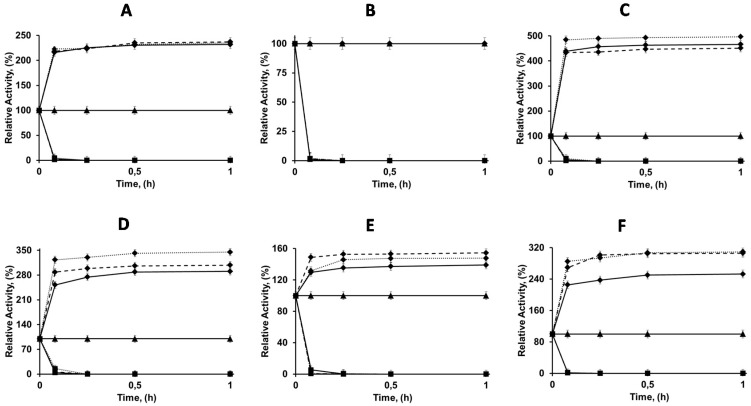
Immobilization courses of different lipases on several octyl agarose supports, OC (Dotted line), OCEDA (Solid black line) and OCHDA (Dashed line). Immobilizations were performed in 50 mM buffer sodium acetate at pH 5 and 25 °C. Other specifications are described in Section 2. (**A**) CALA; (**B**) CALB; (**C**) CRL; (**D**) RML; (**E**) TLL and (**F**) LU. Rhombus (suspension), square (Supernatant), triangle (Soluble enzyme).

At pH 5 and in 50 mM sodium acetate, it has been shown that the ion exchange of the enzymes was negligible. Therefore, these conditions were chosen to immobilize the enzymes on the different OC supports. Figure 3 shows the results. The immobilization rates were fairly similar for all enzymes and supports. As discussed in the Introduction, immobilization via interfacial activation tends to produce a certain hyperactivation due to the stabilization of the open form of the lipase [13]. In the case of CALA, the hyperactivation observed after immobilization in all OC supports was similar (2.3-fold). CALB activity was unaltered by immobilization in the three supports, as expected from previous results and the very tiny lid of this enzyme [14]. CRL hyperactivation was slightly higher using OCHDA (4.9-fold) compared to OC (4.6) or OCEDA (4.5). The same was noted using RML; the hyperactivation was slightly higher using OCHDA (3.45 times) than using OCEDA (3.1-fold) or OC (2.9) and for TLL (1.55 using OCHDA and 1.2 using OC). This contradicts the effect of the immobilization of those enzymes on just aminated supports that produced a certain decrease in enzyme activity (Figure 2). The phospholipase LU followed a similar trend, OCHDA and OCEDA allowed an increment of the activity of more than threefold and OC just of 2.5-fold (Figure 3). The incubation under conditions where the ionic interactions are established (pH7, 5 mM sodium phosphate) did not significantly alter the activity of the immobilized enzymes.

**Figure 4:**
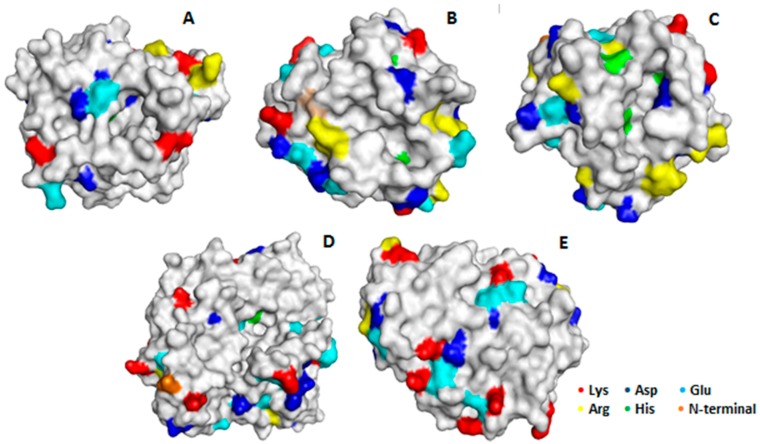
3D surface structure models of lipases showing the face of the active center. (**A**) CALB (PDB Code 1TCA); (**B**) TLL (PDB code 1DT3; (**C**) RML (PDB code 4TGL); (**D**) CRL (PDB code 1CRL); (**E**) CALA (PDB code 3GUU). The 3D structures were obtained from the Protein Data bank (PDB) and displayed using Pymol version 0.99 (DeLano Scientific LLC, Philadelphia, PA, USA).

The results showed that even though the immobilization on the three OC supports should follow a similar mechanism, the further interactions between enzyme and support produced a slight increment on the hyperactivation effect achieved via interfacial activation in monofunctional octyl supports (Figure 3), in contrast with the negative effects that the anion exchange immobilization produced on the enzyme activity (Figure 2). This is a nice example that shows that the immobilization on heterofunctional supports may avoid some of the disadvantages of the use of monofunctional supports, perhaps by changing the orientation of the enzyme and in that way altering the enzyme surface that is in contact with the support. While using monofunctional amino supports the enzyme molecules are immobilized by the area that is the richest one in available anion groups, using the heterofunctional octyl-amino supports, the immobilization is via the area where the active center is located, and this is the only zone of the enzyme surface where it can interact with the support. Figure 4 shows the structure and distribution of anionic and cationic groups of the enzymes when this is available (LU structure is not available). All enzymes have a number of anion residues that may interact with the support after the immobilization via interfacial adsorption, taking advantage of the support surface proximity. However, all of them has also many cation groups in that area.

### 2.3. Confirmation of the Mixed Adsorption of the Different Enzymes and Reversibility of the Immobilization

All studied enzymes could be desorbed from the EDA and HDA supports using 500 mM sodium phosphate at pH 5 or 7. Moreover, CRL, RML, CALA and CALB could be desorbed from the OC support using 1% Triton X-100 at pH 5 or 7. The activities of supernatants and suspensions were used to verify the desorption of the immobilized enzymes. TLL was inactivated by this detergent and LU was so strongly immobilized that even using 2% of detergent, the desorbed enzyme from OC supports was negligible. Thus, the desorption of these two enzymes could not be analyzed. CRL, RML, CALA and CALB were not desorbed, from neither OCEDA nor OCHDA, using 500 mM of sodium phosphate at pH 5 or 7, nor using 1% Triton X-100 at pH 5 or 7, suggesting that the immobilization was not just via interfacial activation. However, the simultaneous use of high buffer concentration and Triton X-100 at pH 5 or 7 allowed us to desorb most of the adsorbed enzyme molecules. This confirmed that, at least for these four enzymes, all enzyme molecules were immobilized via interfacial activation and via ion exchange adsorption. Moreover, it should be remarked that neither CALB nor CALA were fully immobilized in the monofunctional amino supports, while now not enzyme was desorbed using only Triton X-100 from both octyl-amino supports. By incubating at 50 °C the immobilized enzymes in 1% Triton X-100/500 mM sodium phosphate, or 1% SDS or 1% CTAB, all enzyme molecules could be released to the medium and the supports could be reused for 3 successive adsorption/desorption experiments without detecting any change in the support performance: immobilization rate, enzyme activity and stability were similar in all cycles.

### 2.4. Thermal Stabilities of the Differently Immobilized Lipases

Although the immobilization of lipases on the new supports is via interfacial adsorption plus ionic exchange, the effect of this on enzyme stability is unpredictable. In fcat, as a general rule, an inert surface in the supports will be advantageous regarding enzyme stability, as the promotion of any enzyme-support uncontrolled interaction may lead to enzyme inactivation [57].

**Figure 5:**
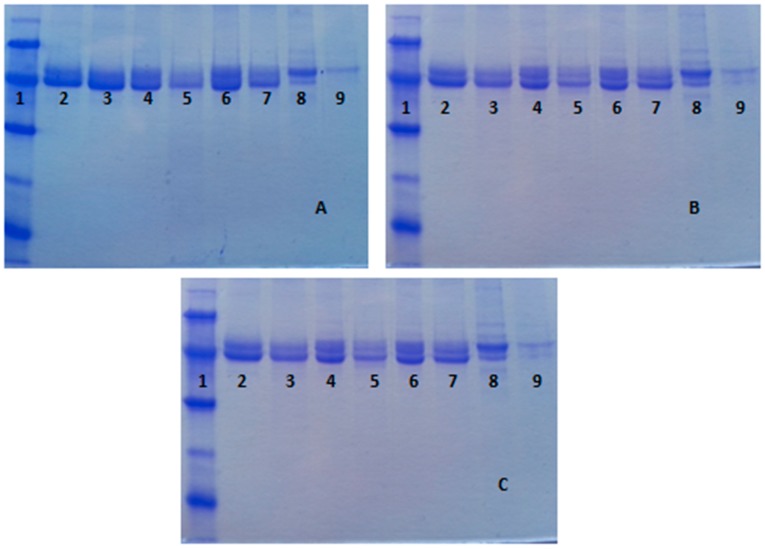
SDS-PAGE analysis of different CALA preparations after thermal inactivation at different pH values. Experiments have been performed as described in Section 2. (**A**) pH 5, (inactivated at 80 °C); (**B**) pH7 (inactivated at 80 °C) and (**C**) pH 9 (inactivated at 70 °C). Other specifications are described in Section 2 or in the Table 1. Lane 1: Low Molecular Weight Marker, Lane 2: OC, Lane 3: OC incubated at pH 5, Lane 4: OCEDA, Lane 5: OCEDA incubated at pH 5, Lane 6: OCHDA, Lane 7: OCHDA incubated at pH 5, Lane 8: EDA and Lane 9: EDA incubated at pH 5.

Therefore, we have analyzed the stabilities of OC, EDA, HDA, OCEDA and OCHDA of the six enzymes at different pH values (from 5 to 9) looking to reinforce or decrease the ionic interactions between enzyme and support. At pH 5 the enzyme and both supports have the highest cationic nature, being the number of negative interactions high. At pH 7, using EDA the cationic nature of the support is reduced, while using HAD the support cationic nature almost does not change. At this pH, the enzymes acquired anionic character. At pH 9, using EDA, the support lost the cation from the primary amino group, while the enzyme has a further increase in its anionic character (see Figure 4). Table 1 shows the half-lives of the different preparations. OC-CALA was the most stable preparation under almost all conditions. Only OCEDA-CALA showed a higher stability at pH 9. CALA immobilized on the new heterofunctional supports were more stable than the only ionic exchanged enzymes, but were 3–4 fold less stable than the OC-CALA. The SDS-PAGE analysis of the different preparations before and after enzyme inactivation under different conditions showed that the heterofunctional supports released more CALA that the OC support (see Figure 5). Apparently, the effect of surface hydrophilization has more relevance than the effect of the ion bonds formed in the enzyme retention. This explained the bad results in terms of stabilization.

**Figure 6:**
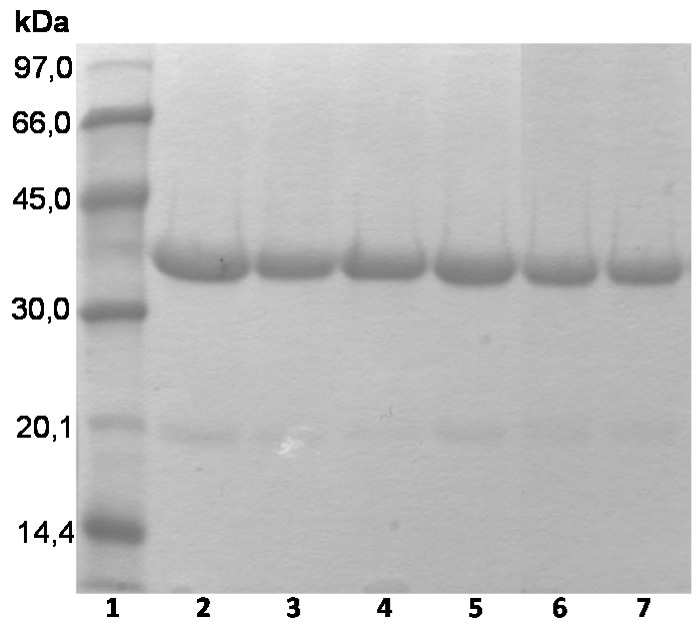
SDS-PAGE analysis of different CALB preparations after thermal inactivation in 5 mM buffer sodium phosphate at pH 7 and 65 °C. Experiments have been performed as described in Section 2. Lane 1: Low Molecular Weight Marker, Lane 2: OC, Lane 3: OC inactivated, Lane 4: OCEDA, Lane 5: OCEDA inactivated, Lane 6: OCHDA, Lane 7: OCHDA inactivated.

In the case of CALB, the highest stability was obtained using OC support at pH 5 and 7 (although OCCALB had a stability very near to that of OCEDA-CALB at pH 7) while at pH 9 the highest stability among the OC supports was observed using OCEDA (by 5-fold) followed by OCHDA (3 times). This occurred even when the enzyme remained adsorbed on the heterofunctional supports after inactivation, while it was partially released from the OC support (see Figure 6). Using CRL, the heterofunctional supports gave lower stabilities at pH 5 and 7 than OC-CRL, while the highest stability was obtained using the just ionically exchanged enzyme. Only the stability of OCHDA-CRL could compare to the stability of the just ionically adsorbed enzyme at pH 9. This showed that the ionic interaction of the enzyme with the support may have very different effects depending on the enzyme orientation and other groups presented in the support. In the case of RM; the stability was not improved using the heterofunctional supports, with a clear drop in the values compared to OC supports; while the just ionically exchanged enzymes were the most stable ones at pH 5 and 7, and the second ones at pH 9. EDA-RML was more stable than HDA-RML.

**Figure 7:**
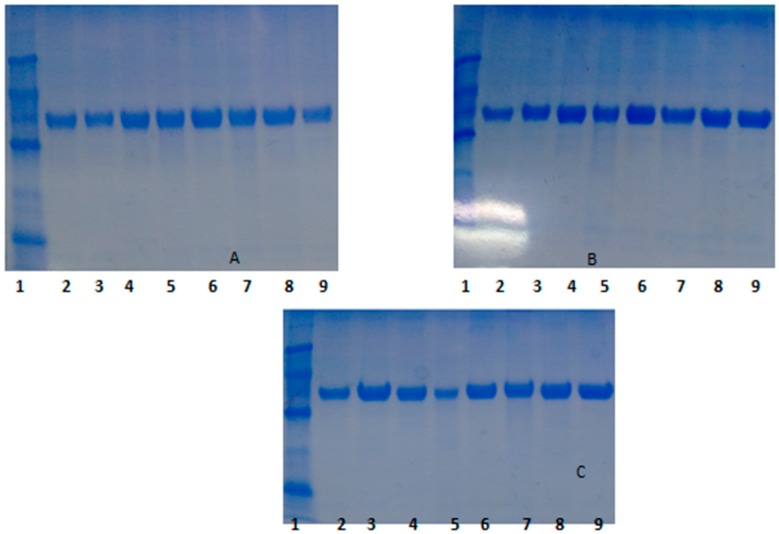
SDS-PAGE analysis of different LU preparations after thermal inactivation at different pH and temperature values. (**A**) pH 5 and 50 °C; (**B**) pH 7 and 50 °C; (**C**) pH 9 and 45 °C. Experiments have been performed as described in Section 2 or in Table 1. Lane 1: Low Molecular Weight Marker, Lane 2: OC, Lane 3: OC inactivated, Lane 4: OCEDA, Lane 5: OCEDA inactivated, Lane 6: OCHDA, Lane 7: OCHDA inactivated, Lane 8: EDA and Lane 9: EDA inactivated.

The case of TLL is more complex. At pH 5, all preparations have similar stability, excluding OC-TLL, that is the least stable. At pH 7, OC-TLL is the most stable, followed by OCHDA-TLL, being OCEDA-TLL the least stable one. At pH 9, OCEDA-TLL is the most stable, followed by OC-TLL and OCHDA-TLL. The ionization of the primary amino group on the support and of the protein surface seems to play a critical role on the stability of this enzyme immobilized on the heterofunctional supports. The highest stability of LU is obtained when immobilized on the ion exchanger supports, and the OC is the least stable one. The analysis of the SDS-PAGE gels obtained before and after enzyme inactivation showed than in this case the enzyme remained immobilized on the support if immobilized on the heterofunctional supports, while it was desorbed from the OC support (Figure 7).

Thus, the use of these heterofunctional supports seemed to have a general negative effect on the thermostability of the enzymes (except at pH 9 for some enzymes, and it is the case of LU and TLL) compared to the OC supports. This could be caused by the promotion of some undesired enzyme-support interactions during protein conformational changes throughout inactivation unfolding that could stabilize incorrect conformations. 

In some cases (e.g., CALB and LU), the use of the heterofunctional supports reduced the enzyme desorption during thermal inactivation. However, some enzymes seemed to be more easily desorbed from the support using the heterofunctional supports than just using OC supports, suggesting that the hydrophilization of the support may be more relevant than the establishment of some few ionic bonds to keep the enzyme immobilized at high temperatures.

### 2.5. Solvent Stabilities of the Differently Immobilized Lipases

Table 2 shows the results obtained in the inactivation of the different lipase preparations using solvents at concentrations where some reliable results could be obtained after some preliminary assays.

**Figure 8:**
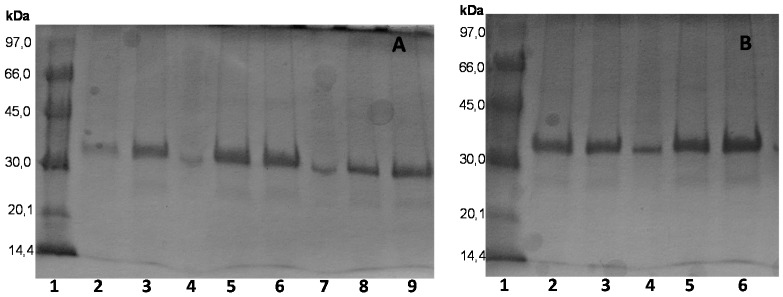
SDS-PAGE analysis of different TLL biocatalysts preparations after inactivation in the presence of DMSO (60% *v*/*v*) in buffer TrisHCl at pH 7 and 30 °C. Experiments have been performed as described in Section 2. (**A**) Lane 1: Low Molecular Weight Marker; Lane 2: OC inactivated, Lane 3: OC, Lane 5: OCEDA inactivated, Lane 6: OCEDA, Lane 8: OCHDA inactivated, Lane 9: OCHDA; (**B**) Lane 1: Low Molecular Weight Marker; Lane 2: EDA inactivated, Lane 3: EDA, Lane 4: OC, Lane 5: HDA inactivated, Lane 6: HDA.

CALA preparations were quite stable and 80% DMSO was required to inactivate the immobilized enzyme. OC-CALA was more stable than the two new preparations, by a 6–8-fold factor, being the least stable ones the just ionically exchanged biocatalysts. CALB was also very stable in organic medium and offered similar half-lives in the three OC supports. Using CRL, the enzyme solvent stability was lower and only 40% acetonitrile was used. The stabilities were similar for the three monofunctional supports, while the new heterofunctional supports offered a lower stability (around 60%). OCRML stability doubled that of the heterofunctional RML biocatalysts on 25% acetonitrile. However, TLL was far more stable on the new heterofunctional supports than on OC supports, although the most stable preparations were the ones prepared by just ionic adsorption. The SDS-PAGE gels of the in activated enzymes (Figure 8) confirmed that the enzyme remained immobilized on OCEDA-TLL and OCHDA-TLL, while it was almost fully desorbed from OC-TLL. LU presented the highest stability after immobilization on the heterofunctional supports, compared to the monofunctional ones.

### 2.6. Reuse of OCEDA-CALB in Hydrolysis of Triacetin to Produce 1,2-Diacetin

**Figure 9:**
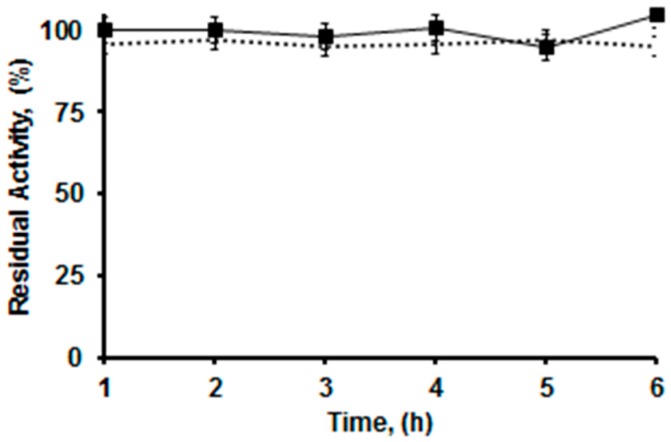
Reuses of OCEDA-CALB in the production of 1,2-diacetin by hydrolysis of triacetin. The experimental conditions are described in Methods section. Solid lane: relative activity (initial activity was taken as 100). Dashed line: 1,2-diacetin yield.

OCEDA-CALB was used in six consecutive reaction cycles for the production of 1,2-diacetin (a process that shows the regioselectivity and specificity of the enzyme, see Methods section) by hydrolysis of triacetin at pH 5 in the presence of 20% acetonitrile. The activity of the preparation was mantained during the 6 cycles and 93%–95% 1,2-diacetin could be obtained after just one hour of reaction time (Figure 9), similar to the results obtained using the OCCALB preparation.

## 3. Discussion

The use of heterofunctional amino/octyl supports prevents the enzyme desorption from the biocatalysts in the presence of non-ionic detergents, while keeping the reversibility of the immobilization. The first immobilization is via interfacial activation, and later some ionic bridges are established between the enzyme and the support, avoiding enzyme desorption when the hydrophobic interactions were broken. This mixed adsorption has complex effects on enzyme stability, sometimes increasing it, sometimes decreasing it.

The effect of the introduction of amino groups on octyl agarose on the stability of lipases is quite diverse, depending strongly on the enzyme. For LU and TLL, the use of OC-amino supports offered clear advantages in terms of stability, while for the other four enzymes the results turn out to be negative (except at some pH values). Generally, when the ionization of the primary amino group decrease (e.g., pH 9 using OCEDA), the results are better than when both amino groups are fully ionized. Curiously, this occurred even in the cases where the enzymes immobilized on monofunctional amino supports were the most stable preparations (e.g., RML). The explanation may be diverse. Although all of the enzymes have some anionic groups on the face of the active center (Figure 4), many enzymes have also many cationic groups. The case where a highest stabilization was achieved was TLL; and that enzyme has a clear crown of anion groups surrounding the active center. 

The presence of many enzyme cation groups near the support surface may render the effect of support surface hydrophilization (that may weaken enzyme adsorption) more relevant than the effect of ion exchange bridges on enzyme stability, and might explain that, under thermal inactivation, enzyme desorption from the support is even higher than using just octyl supports in many instances. Other possibility to explain the stability results is the promotion of undesired enzyme supports interactions during enzyme inactivation (the enzyme will suffer partial unfolding) that can fix incorrect conformations. These conformational changes in enzyme structure may be different depending on the inactivation pH values and may also justify the different relative stability under different pH values [58].

Solutions to improve the results are varied. If maintaining the reversibility is desired, the number of anion groups in the active center face may be increased via site-directed mutagenesis to reinforce the ion adsorption of the immobilized enzyme [59]. Other possibility is the modification of the support, e.g., adding anion groups [60], or even mixed cation/anion groups [61]. Some authors suggest that enzyme refolding of immobilized enzymes is easier if the support has the same ion nature of the enzyme, this may help in improving enzyme activity recovery during activity determination under mild conditions [62,63,64,65,66].

Chemical modification (e.g., succinylation) of the primary amino groups did not seem very adequate, due to the relatively poor amount of Lys residues in this area for most enzymes. Other alternative, that will eliminate the reversibility of the immobilization process, may be the treatment of the immobilized enzymes with some crosslinking reagents, like glutaraldehyde. This will modify the enzyme (in the case of lipases this modification may be positive in terms of activity and stability) but also will permit the covalent immobilization [67]. However, this will eliminate the reversibility, one of the objectives of this paper.

Considering that the enzyme support interactions may alter enzyme conformation (as showed by the changes in activity and changes in secondary structure) [58], these supports may be a new opportunity to increase the library of immobilized biocatalysts of a particular lipase, of interest if the objective is the modulation of the enzyme properties [68].

## 4. Materials and Methods

### 4.1. Materials

Solutions of lipase A from *Candida antarctica* (CALA) (19.9 mg of protein per mL), lipase B from *Candida antarctica* (CALB) (10.5 mg of protein per mL), lipase from *Thermomyces lanuginosus* (TLL) (36 mg of protein per mL), lipase from *Rhizomucor miehei* (RML) (13.7 mg of protein per mL) and the phospholipase Lecitase Ultra (LU) (16 mg of protein per mL) were a kind gift from Novozymes (Madrid, Spain). Octyl-agarose beads support was obtained from GE Healthcare (Madrid, Spain)). Hexamethylenediamine (HDA), ethylenediamine (EDA), *p*-nitrophenyl butyrate (*p*-NPB), and lipase from *Candida rugosa* (CRL) (in powder form, 40% of protein content) were from Sigma Chemical Co. (St. Louis, MO, USA). All reagents and solvents were of analytical grade. Glyoxyl (GLX) and octyl-glyoxyl (OCGLX) were prepared as described in [34].

### 4.2. Standard Determination of Enzyme Activity

This assay was performed by measuring the increase in absorbance at 348 nm (isosbestic point of *p*-nitrophenol (*p-*NP)) produced by the released *p-*NP in the hydrolysis of 0.4 mM *p*-NPB in 25 mM sodium phosphate at pH 7.0 and 25 °C (ε under these conditions is 5150 M^−1^·cm^−1^). This substrate was selected because it is soluble in homogenous media and prevents the use of surfactants that can alter the behavior of the free or immobilized lipases. To initialize the reaction, a volume of 50–100 μL of lipase solution or suspension was added to 2.5 mL of substrate solution. One international unit of activity (U) was defined as the amount of enzyme that hydrolyzes 1 μmol of *p*-NPB per minute under the conditions described previously. Protein concentration was determined using Bradford’s method [69] and bovine serum albumin was used as the reference.

### 4.3. Preparation of Amino Supports

The preparation is schematized in Figure 1. The modification is a well-established protocol based in full modification of glyoxyl groups with aminated compounds, in this case we have 25 µmol aldehyde groups per g of wet OCGLX and 30 µmol aldehyde groups per g of wet of GLX support [36,43,70,71,72,73]. 20 g of GLX or OCGLX supports were resuspended in 100 mL of 2 M hexamethylendiamine (HDA) or ethylendiamine (EDA) solution at pH 10.1 to obtain the respective aminated supports (ECA, OCEDA, HDA or OCHDA). The suspensions containing the supports and the amino solutions were gently stirred for 3 h at 25 °C. To end the support modification, we added solid sodium borohydride until reaching a concentration of 10 mg/mL, and submitted to gentle stirring for 30 additional minutes. This treatment reduces reversible Schiff’s bases to very stable secondary amino bonds, and unreacted aldehyde groups to fully inert hydroxy groups [43]. Finally, the four reduced supports were filtered, washed with abundant distilled water and stored at 4 °C. A wet support is defined as the agarose beads with the pores full of aqueous medium, but without interparticle water (dried using a vacuum filter). 

### 4.4. Immobilization of Enzymes

#### 4.4.1. Immobilization of Enzymes on Octyl (OC) and Octyl-Amino (OCHDA and OCEDA) Supports

The immobilization was performed using 1 (for activity and stability studies) or 5 mg (for SDS-PAGE analysis) of protein per g of wet support. The commercial samples of the enzymes were diluted in the corresponding volume of 50 mM sodium acetate at pH 5. Then 20 g of support were added to 200 mL of enzyme solution at 25 °C under gentle stirring. The activities of both supernatant and suspension were followed using *p*-NPB. After immobilization, the suspension was filtered and washed several times with distilled water and stored at 4 °C. In the case of OCHDA and OCEDA, the immobilized enzyme was filtered, washed and resuspended in 5 mM sodium phosphate buffer at pH 7 and 25 °C for a minimum of 12 h, to allow the enzyme-support ionic interaction. 

#### 4.4.2. Immobilization of Enzymes on HDA and EDA Supports 

The immobilization was performed using 1 or 5 mg of protein per g of wet support (see above). The commercial samples of the enzymes were diluted in the corresponding volume of 5 mM sodium phosphate at pH 7. Then, 20 g of support were added to 200 mL of enzyme solution at 25 °C under gentle stirring. The activities of both supernatant and suspension were followed using *p*-NPB. After immobilization, the suspensions were filtered and washed several times with the same buffer of immobilization and stored at 4 °C. 

### 4.5. Thermal Inactivation of Different Enzymatic Preparations

The stability of the different enzyme preparations was determined by re-suspending 1 g of immobilized enzyme in 5 mL of 5 mM sodium acetate at pH 5, sodium phosphate at pH 7 or sodium carbonate at pH 9 at different temperatures. Periodically, samples of the inactivation suspension were withdrawn and the activity was measured using *p-*NPB. Half-lives were calculated from the observed inactivation courses.

### 4.6. Inactivation of Different Enzymatic Preparations by Incubation in Organic Co-Solvents 

A solvent and concentration where the inactivation rate of the biocatalyst permitted to have accurate measurements was chosen for each enzyme. Enzyme preparations were incubated in mixtures of dimethyl sulfoxide (DMSO), acetonitrile (ACN), or 1,4-dioxane/5 mM Tris–HCl at pH 7 and 30 °C. Periodically, samples of the inactivation suspensions were withdrawn and the activities were measured using *p*-NPB (see above). Half-lives were calculated from the observed inactivation courses. The organic co-solvents presented in the samples did not have a significant effect in on the enzyme activity assays (results not shown). 

### 4.7. SDS-PAGE Analysis

SDS-polyacrylamide gel electrophoresis (SDS-PAGE) was performed according to Laemmli [74] using a Miniprotean tetra-cell (BioRad, Alcobendas, Spain), 12% running gel in a separation zone of 9 cm × 6 cm, and a concentration zone of 5% polyacrylamide. One hundred milligrams of the immobilized enzyme samples were resuspended in 1 mL of rupture buffer (2% SDS and 10% mercaptoethanol), boiled for 10 min and then a 20 μL aliquot of the supernatant was used in the experiments. Gels were stained with Coomassie brilliant blue. Low molecular weight markers (10–200 kDa) from Fermentas (Alcobendas, Spain) were used. 

### 4.8. Hydrolysis of Triacetin

The production of 1,2-diacetin is an interesting use of these biocatalyst, requiring the presence of organic solvent and an acid pH to improve yields and prevent acyl migration [75,76]. Solutions of 7 mM triacetin in 20 mM sodium acetate/acetonitrile (20% *v*/*v*) were prepared, and their pH values were adjusted at 5.0 using NaOH. Samples of 0.25 g of wet OCEDA or OC-CALB were added to 40 mL of the triacetin solutions and the reaction suspensions were gently stirred in a shaker at 250 rpm and 22 °C. Periodically, samples were withdrawn, the biocatalyst was discarded by centrifugation and the concentration of reaction products was analyzed by HPLC. Diacetin and triacetin were analyzed using 10% acetonitrile/ 90% water (*v*/*v*) as mobile phase at a flow rate of 1 mL/min and a RP-HPLC (SP 100, Spectra Physics, Alcobendas, Madrid, Spain, coupled with a Spectra Physics SP 8450 UV detector) using a Kromasil C18 (15 cm × 0.46 cm) column, retention volumes were 32.0 mL for triacetin, 5.8 mL for 1,2-diacetin, 4.8 mL for 1,3-diacetin. The pH value decreased during the reaction due to the production of acetic acid, but the pH was not controlled to avoid risks of acyl migration, the final reaction pH was 4.35.

## Figures and Tables

**Table 1:** Half-lives (in minutes) of the different biocatalysts under different experimental conditions.

Biocatalyst	Experimental Conditions		
	pH 5.0	pH 7.0	pH 9.0
OCCALA	360 ± 10	60 ± 5	42 ± 4
OCEDACALA	240 ± 10	18 ± 2	90 ± 9
OCHDACALA	120 ± 11	15 ± 1	24 ± 3
EDACALA	120 ± 8	12 ± 1	15 ± 2
HDACALA	120 ± 9	9 ± 1	12 ± 1
OCCALB	120 ± 10	300 ± 18	60 ± 5
OCEDACALB	18 ± 2	300 ± 20	300 ± 31
OCHDACALB	12 ± 1	108 ± 9	180 ± 19
OCCRL	180 ± 11	60 ± 4	48 ± 5
OCEDACRL	12 ± 1	18 ± 3	12 ± 2
OCHDACRL	12 ± 1	18 ± 2	360 ± 14
EDACRL	240 (100%) *	240 (100%) *	420 ± 21
HDACRL	240 (100%) *	240 (100%) *	420 ± 30
OCRML	360 ± 20	330 ± 32	720 ± 75
OCEDARML	210 ± 15	108 ± 12	29 ± 1
OCHDARML	60 ± 7	48 ± 4	45 ± 5
EDARML	360 (100%) *	360 (100%) *	120 ± 4
HDARML	360 (100%) *	360 (70%) *	18 ± 3
OCTLL	300 ± 25	480 (80%) *	72 ± 6
OCEDATLL	1440 (80%) *	10 ± 1	180 ± 11
OCHDATLL	1440 (80%) *	228 ± 15	90 ± 10
EDATLL	1440 (80%) *	60 ± 6	30 ± 2
HDATLL	1440 (80%) *	60 ± 5	24 ± 3
OCLU	18 ± 1	24 ± 5	60 ± 11
OCEDALU	240 ± 13	180 ± 12	600 ± 90
OCHDALU	240 ± 12	180 ± 21	600 ± 80
EDALU	180 (80%) *	180 (100%) *	1440 (100%) *
HDALU	180 (100%) *	180 (100%) *	1440 (100%) *

CALA (pH 5.0 at 80 °C, pH 7.0 at 45 °C and pH 9.0 at 45 °C); CALB (pH 5.0 at 80 °C, pH 7.0 at 65 °C and pH 9.0 at 60 °C); CRL (pH 5.0 at 60 °C, pH 7.0 at 55 °C and pH 9.0 at 40 °C); RML (pH 5.0 at 50 °C, pH 7.0 at 80 °C and pH 9.0 at 70 °C); TLL (pH 5.0 at 60 °C, pH 7.0 at 65 °C and pH 9.0 at 65 °C); LU (pH 5.0 at 50 °C, pH 7.0 at 50 °C and pH 9.0 at 45 °C); * The enzyme activity did not reach the half-live during the assay-time.

**Table 2:** Half-lives (in minutes) of the different biocatalysts in the presence of different concentrations of organic solvents in buffer TrisHCl at pH 7 and incubated at 30 °C.

Biocatalyst	Experimental Conditions			
	ACN 25%	DMSO 80%	ACN 40%	Dioxane 60%
OCCALA	-	210 ± 18	-	-
OCEDACALA	-	48 ± 3	-	-
OCHDACALA	-	24 ± 3	-	-
EDACALA	-	15 ± 1	-	-
HDACALA	-	15 ± 2	-	-
OCCALB	-	150 ± 11	-	-
OCEDACALB	-	162 ± 10	-	-
OCHDACALB	-	150 ± 10	-	-
OCCRL	-	-	30 ± 2	-
OCEDACRL	-	-	18 ± 1	-
OCHDACRL	-	-	18 ± 2	-
EDACRL	-	-	28 ± 3	-
HDACRL	-	-	42 ± 3	-
OCRML	24 ± 2	-	-	-
OCEDARML	12 ± 1	-	-	-
OCHDARML	12 ± 2	-	-	-
EDARML	12 ± 1	-	-	-
HDARML	18 ± 2	-	-	-
OCTLL	-	-	-	348 ± 33
OCEDATLL	-	-	-	1440 (80%) *
OCHDATLL	-	-	-	1440 ± 100
EDATLL	-	-	-	1440 (100%) *
HDATLL	-	-	-	1440 (100%) *
OCLU	15 ± 2	-	-	-
OCEDALU	102 ± 9	-	-	-
OCHDALU	84 ± 5	-	-	-
EDALU	18 ± 1	-	-	-
HDALU	30 ± 2	-	-	-

* The enzyme activity did not reach the half-live during the assay-time.

## Abbreviations

The following abbreviations are used in this manuscript:

OC	OCTYL SUPPORT Multidisciplinary Digital Publishing Institute
EDA	DIETHYLENAMINE MODIFIED SUPPORT
HDA	DIHEXYENAMINE MODIFIED SUPPORT
OCEDA	OCTYL-DIETHYLENAMINE MODIFIED SUPPORT
OCHDA	OCTYL-DIHEXYENAMINE MODIFIED SUPPORT
